# Perioperative variation in serum FGF-23 level and its correlation with MACCE risk in unprotected left main coronary artery disease patients receiving coronary artery bypassing grafting

**DOI:** 10.3389/fsurg.2022.937342

**Published:** 2022-09-05

**Authors:** Fanshun Wang, Runhua Ma, Chunsheng Wang

**Affiliations:** Department of Cardiac Surgery, Zhongshan Hospital, Fudan University, Shanghai, China

**Keywords:** unprotected left main coronary artery disease, coronary artery bypass grafting, fibroblast growth factor 23, major adverse cardiac and cerebrovascular event, risk factor

## Abstract

**Objective:**

Fibroblast growth factor-23 (FGF-23) mediates vascular endothelial injury, inflammatory infiltration, and atherosclerosis, which could reflect major adverse cardiac and cerebrovascular event (MACCE) risk in several cardiovascular diseases. This study aims to further investigate the perioperative change of FGF-23, as well as its association with clinical characteristics and MACCE risk in unprotected left main coronary artery disease (ULMCAD) patients receiving coronary artery bypass grafting (CABG).

**Methods:**

A total of 226 ULMCAD patients who underwent CABG were enrolled. Serum samples of the patients were collected on the day before CABG, the third day (D3) after CABG, and at discharge; then, the FGF-23 level was determined by enzyme-linked immunosorbent assay. The MACCE rate was recorded during a median follow-up of 25.5 (range: 2.0–46.0) months.

**Results:**

The median, interquartile range (IQR), and range of FGF-23 level in ULMCAD patients receiving CABG were 717.0, 582.5–869.8, and 407.0–1765.0 pg/ml, respectively. FGF-23 level was increased in patients with both previous heart failure (*P *= 0.046) and chronic renal failure (*P *= 0.009) compared to those without. FGF-23 level increased from before surgery [median (IQR): 712.5 (574.5–879.8) pg/ml] to D3 [median (IQR): 844.0 (666.0–1072.5) pg/ml], then declined at discharge [median (IQR): 764.5 (569.3–986.8) pg/ml] (*P *< 0.001). Meanwhile, the preoperative FGF-23 level (*P *= 0.028), but not the FGF-23 level at discharge (*P *= 0.067) was positively correlated with the cumulative MACCE rate. Multivariable Cox's analyses found that preoperative FGF-23 level could independently predict cumulative MACCE rate [*P *= 0.015, hazards ratio (HR) = 2.940].

**Conclusion:**

Preoperative FGF-23 level predicts higher MACCE risk in ULMCAD patients undergoing CABG surgery.

## Introduction

Unprotected left main coronary artery disease (ULMCAD) is a highly lethal cardiovascular disease, which is defined as more than 50% stenosis of the left coronary artery, and without patent bypass graft to the left branches (1). In recent decades, remarkable progress has been made in coronary revascularization of ULMCAD and coronary artery bypass grafting (CABG) is one of the revascularization strategies that has been considered the main treatment modality for ULMCAD patients (2–4). However, even after CABG surgery, major adverse cardiac and cerebrovascular event (MACCE) occurs frequently in patients with ULMCAD, which brings a huge challenge to clinicians (5–7). Consequently, it is crucial to find potential biomarkers to early assess the risk of MACCE and thereby improve the management of ULMCAD patients undergoing CABG.

Fibroblast growth factor-23 (FGF-23) is a bone-derived hormone, which is a fundamental regulator of phosphate and vitamin D homeostasis (8, 9). Recently, studies have shown that FGF-23 has a regulatory role in the pathological process of various cardiovascular diseases, and it has the potential to become a clinical biomarker for predicting the risk of MACCE (10–12). For example, FGF-23 regulates cardiac hypertrophy by acting directly on FGF receptor 4 (FGFR4), while FGFR4 activation requires klotho as a cofactor (13). Meanwhile, serum FGF-23 is associated with major adverse cardiovascular events (MACE) risk in patients undergoing coronary angiography (14). Moreover, plasma FGF-23 is a risk factor for MACCE in patients with end-stage renal disease (ESRD) receiving continuous ambulatory peritoneal dialysis (CAPD) (11). Based on the above considerations, it could be hypothesized that FGF-23 might also be associated with the risk of MACCE in ULMCAD patients. However, no research found this to be the case.

Therefore, this study aims to investigate the perioperative variation of serum FGF-23 and its linkage with clinical features as well as MACCE risk in ULMCAD patients receiving CABG.

## Methods

### Patients

This study consecutively included 226 ULMCAD patients who received their first-ever CABG from May 2017 to December 2020. Patients who met the following criteria were eligible for inclusion: (a) angiographical diagnosis of ULMCAD, which was defined as the narrow of left main coronary artery >50% and without patent bypass grafts to its branches (15); (b) aged over 18 years; (c) planned to receive CABG; (d) willing to provide peripheral blood (PB) samples; (e) willing to perform follow-up visit required by the protocol. The exclusion criteria were as follows: (a) had ST-elevation myocardial infarction within 24 h; (b) had a high risk of CABG or had contraindications of CABG; (c) had a prior history of CABG; (d) during pregnant or lactating. The study was permitted by Ethics Committee. The written informed consents were collected from patients or guardians.

### Data collection

Clinical characteristics of the eligible patients were recorded after recruitment, which included demographics, hypertension, hyperlipidemia, diabetes, smoke status, family history of coronary artery disease (CAD), previous myocardial infarction, previous heart failure, previous stroke, previous chronic lung disease, previous chronic renal failure, previous PCI, clinical presentation, left ventricular ejection fraction (LVEF), disease extent; distal bifurcation involvement, and right CAD involvement.

### Samples collection and detection

PB samples were collected from the eligible patients on the day before CABG (*n* = 226), 3 days (D3) after CABG (*n* = 219), and on the day of discharge (*n* = 217). Then, the collected PB samples were centrifuged at 3,500 revolutions per minute for 10 min to isolate serum samples, and the serum samples were stored at −80 °C for further detection. Sequentially, serum samples were used to measure FGF-23 level by enzyme-linked immunosorbent assay (ELISA) using Human FGF-23 DuoSet ELISA (DY2604-05, sensitivity: 8.0 pg/ml, Bio-Techne China Co. Ltd., Minneapolis, MN, USA). The experimentation was performed in strict accordance with the manufacturer's instructions. In the analysis, the median value of FGF-23 level was used to classify the patients as high group and low group.

### Coronary artery bypass grafting operation

According to the 2011 CABG Guideline issued by the American College of Cardiology Foundation (16), CABG procedures were carried out with standard techniques. Briefly, a midline thoracotomy was performed, and the left internal mammary artery was separated from the left heart outside the pleura, then the great saphenous vein or radial artery was grafted. Sequentially, distal vascular anastomosis was constructed with the choice of on- or off-pump.

### Follow-up and assessment

Patients were followed up continuously by clinic visits every 3–6 months until 31 December 2021, and the median follow-up period was 25.5 months. Based on follow-up, major adverse cardiac and cerebrovascular event (MACCE) was recorded, which was defined as the composite of death for any reason, myocardial infarction, stroke, and repeat revascularization (17). In addition, the cumulative MACCE rate was calculated for evaluation. During the study period, approximately 31 patients were lost to follow-up, and we used censored data to calculate the accumulating MACCE rate.

### Statistics

In order to avoid the deviation caused by short follow-up, patients lost to follow-up within 1 year were excluded from the analysis. Statistical analysis was completed by SPSS V.22.0 (IBM Corp., Armonk, NY, USA), and figure plotting was fulfilled by GraphPad Prism V.7.02 (GraphPad Software Inc., USA). The correlation of FGF-23 level with clinical characteristics was estimated using the Kruskal–Wallis H rank sum test or Wilcoxon rank sum test. Association of FGF-23 level with cumulative MACCE rate was measured using the Kaplan-Meier method and log-rank test. Change in FGF-23 level over time was analyzed using the Friedman test, followed by *post hoc* comparisons with the Bonferroni test. Factors affecting the cumulative MACCE rate were determined using forward-stepwise multivariable Cox's proportional hazards regression analyses (the factors shown in Table 1 were included), and the independent prognostic factors were further determined by the receiver operating characteristic (ROC) curve. Statistical significance was declared as a two-side *P* value <0.05.

**Table 1:** Patient's characteristics.

Items	ULMCAD patients (*N* = 226)
Age (years), mean ± SD	64.8 ± 7.6
Gender, no. (%)	
Female	49 (21.7)
Male	177 (78.3)
BMI (kg/m^2^), mean ± SD	25.1 ± 2.9
Hypertension, no. (%)	138 (61.1)
Hyperlipidemia, no. (%)	122 (54.0)
Diabetes, no. (%)	55 (24.3)
Current smoker, no. (%)	62 (27.4)
Family history of CAD, no. (%)	42 (18.6)
Previous myocardial infarction, no. (%)	58 (25.7)
Previous heart failure, no. (%)	8 (3.5)
Previous stroke, no. (%)	12 (5.3)
Previous chronic lung disease, no. (%)	9 (4.0)
Previous chronic renal failure, no. (%)	10 (4.4)
Previous PCI, no. (%)	26 (11.5)
Clinical presentation, no. (%)	
Unstable angina	119 (52.7)
Stable angina	107 (47.3)
LVEF <50%, no. (%)	
No	185 (81.9)
Yes	41 (18.1)
Disease extent, no. (%)	
Left main only	7 (3.1)
Left main +1 vessel disease	15 (6.6)
Left main +2 vessel diseases	48 (21.2)
Left main +3 vessel diseases	156 (69.0)
Distal bifurcation involvement, no. (%)	
No	67 (29.6)
Yes	159 (70.4)
Right CAD involvement, No. (%)	
No	52 (23.0)
Yes	174 (77.0)

ULMCAD, unprotected left main coronary artery disease; SD, standard deviation; BMI, body mass index; CAD, coronary artery disease; PCI, percutaneous coronary intervention; LVEF, left ventricular ejection fraction.

## Results

### Patient's characteristics

The mean age of ULMCAD patients was 64.8 ± 7.6 years, with 49 (21.7%) females and 177 (78.3%) males. Regarding the clinical characteristics of ULMCAD patients, there were 119 (52.7%) patients with unstable angina and 107 (47.3%) patients with stable angina. Meanwhile, there were 185 (81.9%) patients with LVEF ≥50%, while 41 (18.1%) patients with LVEF <50%. Relating to disease extent, there were 7 (3.1%) patients with left main only, 15 (6.6%) patients with left main +1 vessel diseases, 48 (21.2%) patients with left main +2 vessel diseases, and 156 (69.0%) patients with left main +3 vessel diseases. Concerning right CAD involvement, there were 52 (23.0%) patients without right CAD involvement and 174 (77.0%) patients with right CAD involvement. The detailed ULMCAD patients' characteristics are shown in Table 1.

### Serum FGF-23 level distribution

**Figure 1:**
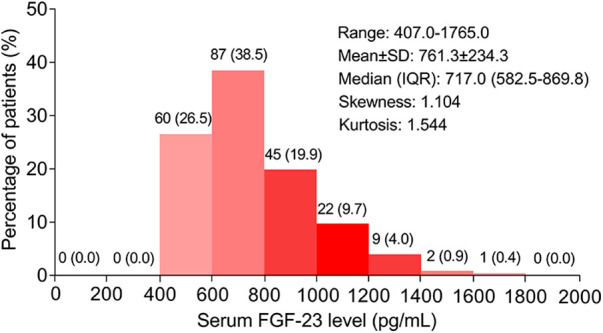
Distribution of serum FGF-23 level in unprotected left main coronary artery disease patients receiving coronary artery bypass grafting .

The ULMCAD patients' serum FGF-23 level ranged from 407.0 to 1765.0 pg/ml, with a mean value of 761.3 ± 234.3 pg/ml and a median [interquartile range (IQR)] value of 717.0 (582.5–869.8) pg/ml. The specific distribution of serum FGF-23 in ULMCAD patients is shown in Figure 1.

### Comparison of FGF-23 levels in patients with different clinical characteristics

FGF-23 level was increased in ULMCAD patients with previous heart failure (*P *= 0.046) by contrast to those without. Meanwhile, FGF-23 was also elevated in ULMACD patients with previous chronic renal failure (*P *= 0.009) compared to those without (Table 2).

**Table 2:** Comparison of FGF-23 level in patients with different clinical characteristics.

Items	Serum FGF-23 level (pg/ml) median (IQR)	*P* value
Age		
<65 years	734.5 (574.5–924.5)	0.315
≥65 years	709.0 (586.3–820.5)	
Gender		
Female	709.0 (608.5–897.5)	0.907
Male	723.0 (572.0–863.5)	
BMI		
<25 kg/m^2^	738.0 (599.0–929.5)	0.186
≥25 kg/m^2^	712.0 (568.0–852.0)	
Hypertension		
No	712.0 (544.5–854.8)	0.252
Yes	726.0 (600.5–892.5)	
Hyperlipidemia		
No	723.0 (571.0–879.5)	0.759
Yes	717.0 (598.5–856.3)	
Diabetes		
No	712.0 (565.0–860.0)	0.128
Yes	750.0 (628.0–892.0)	
Current smoker		
No	710.5 (571.0–858.8)	0.376
Yes	743.0 (606.5–896.5)	
Family history of CAD		
No	712.0 (577.5–858.8)	0.189
Yes	774.0 (592.0–949.8)	
Previous myocardial infarction		
No	723.5 (564.3–858.8)	0.098
Yes	717.0 (651.3–979.8)	
Previous heart failure		
No	713.0 (572.5–861.0)	0.046
Yes	926.0 (670.8–1339.5)	
Previous stroke		
No	713.0 (572.5–861.0)	0.121
Yes	736.5 (670.8–1231.3)	
Previous chronic lung disease		
No	713.0 (574.0–879.0)	0.706
Yes	744.0 (688.5–810.0)	
Previous chronic renal failure		
No	712.5 (571.5–859.8)	0.009
Yes	936.0 (708.8–1368.3)	
Previous PCI		
No	721.0 (568.8–863.0)	0.476
Yes	706.5 (640.3–979.8)	
Clinical presentation		
Unstable angina	692.0 (585.0–878.0)	0.274
Stable angina	742.0 (573.0–860.0)	
LVEF <50%		
No	721.0 (580.0–858.5)	0.307
Yes	709.0 (584.0–1124.5)	
Disease extent		
Left main only	712.0 (623.0–864.0)	0.641
Left main +1 vessel disease	664.0 (544.0–752.0)	
Left main +2 vessel diseases	727.5 (573.5–919.5)	
Left main +3 vessel diseases	731.0 (592.8–858.8)	
Distal bifurcation involvement		
No	696.0 (547.0–857.0)	0.294
Yes	729.0 (597.0–906.0)	
Right CAD involvement		
No	769.0 (573.0–1015.8)	0.096
Yes	712.0 (582.5–848.8)	

FGF-23, fibroblast growth factor-23; IQR, interquartile range; BMI, body mass index; CAD, coronary artery disease; PCI, percutaneous coronary intervention; LVEF, left ventricular ejection fraction.

### Perioperative variation of serum FGF-23 level

**Figure 2:**
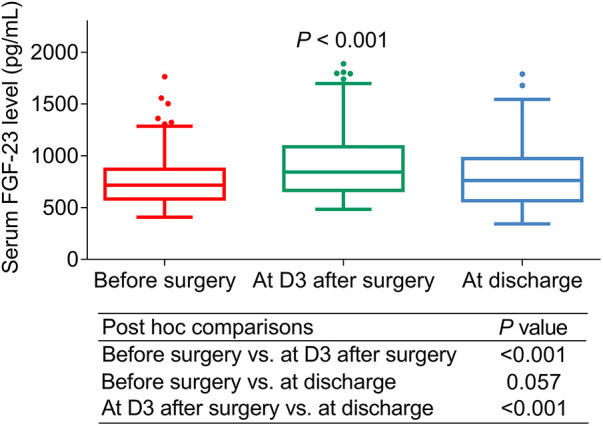
Serum FGF-23 level changed before and after coronary artery bypass grafting (CABG) surgery in unprotected left main coronary artery disease patients receiving CABG.

Serum FGF-23 level in ULMCAD patients showed an upward trend from before surgery [median (IQR) level: 712.5 (574.5–879.8) pg/ml] to D3 after surgery [median (IQR) level: 844.0 (666.0–1072.5) pg/ml], then there was a certain decline at discharge [median (IQR) level: 764.5 (569.3–986.8) pg/ml] (*P *< 0.001). Besides, further multiple comparisons exhibited that the FGF-23 level was increased at D3 after surgery compared to before surgery (*P *< 0.001). Meanwhile, no difference was found between the FGF-23 level before surgery and at discharge (*P *= 0.057). Moreover, the FGF-23 level was reduced at discharge compared with at D3 after surgery (*P *< 0.001) (Figure 2).

### Correlation between FGF-23 (before surgery) level and cumulative MACCE rate

**Figure 3:**
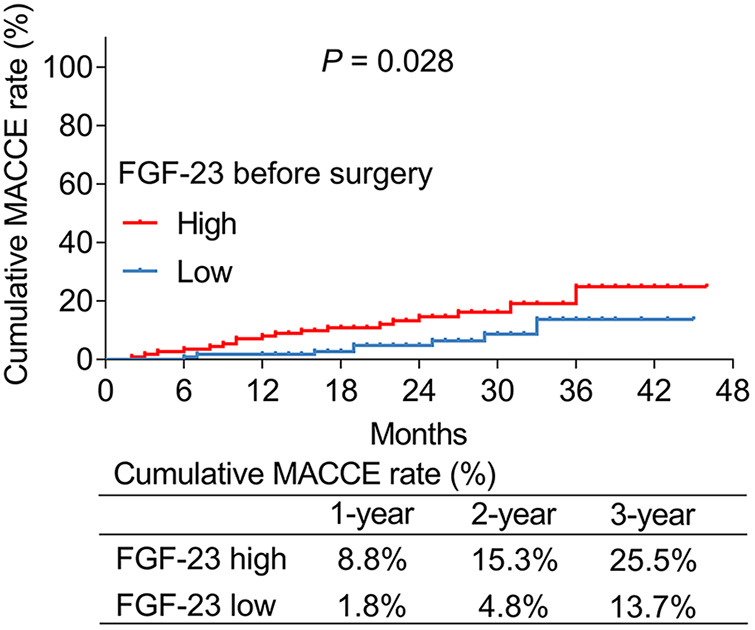
FGF-23 (before surgery) level was positively correlated with cumulative major adverse cardiac and cerebrovascular event rate in unprotected left main coronary artery disease patients receiving CABG.

According to the median value of ULMCAD patients' FGF-23 level before surgery, they were divided into FGF-23 (before surgery) high and low groups. It was found that the cumulative MACCE rate was increased in the FGF-23 (before surgery) high group compared to the FGF-23 (before surgery) low group (*P *= 0.028) (Figure 3). Specifically, the 1-year, 2-year, and 3-year cumulative MACCE rate in FGF-23 (before surgery) high group was 8.8%, 15.3%, and 25.5%, respectively; while 1.8%, 4.8%, and 13.7% in FGF-23 (before surgery) low group, respectively.

### Correlation between FGF-23 (at discharge) level and cumulative MACCE rate

**Figure 4:**
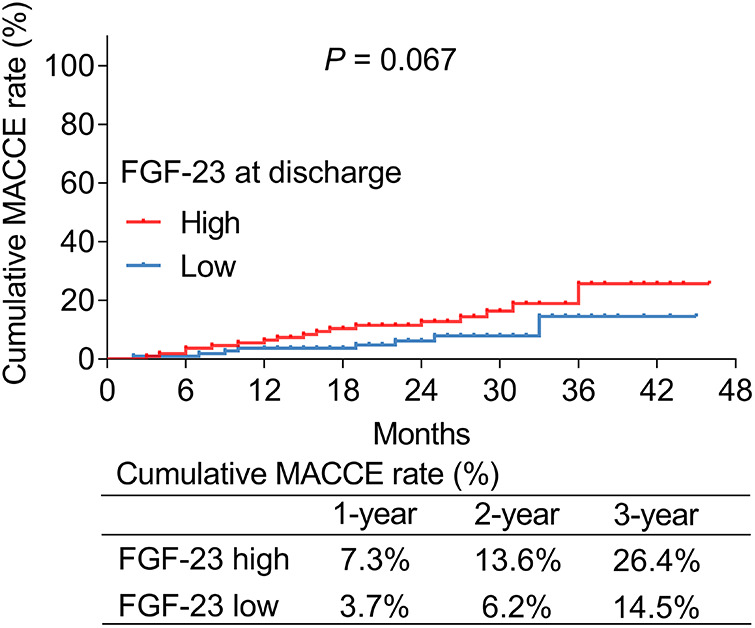
FGF-23 (at discharge) level exhibited a trend to be positively correlated with the cumulative major adverse cardiac and cerebrovascular event rate in unprotected left main coronary artery disease patients receiving coronary artery bypass grafting.

According to the median value of ULMCAD patients' FGF-23 level at discharge, they were divided into FGF-23 (at discharge) high and low groups. It was found that the cumulative MACCE rate was increased slightly in FGF-23 (at discharge) high group compared to FGF-23 (at discharge) low group but did not reach statistical significance (*P *= 0.067) (Figure 4). Particularly, the 1-year, 2-year, and 3-year cumulative MACCE rate in FGF-23 (at discharge) high group was 7.3%, 13.6%, and 26.4%, respectively; but was 3.7%, 6.2%, and 14.5% in FGF-23 (at discharge) low group, respectively.

### Adjustment by multivariate Cox's hazards regression analysis

Multivariate Cox's proportional hazards regression analysis exhibited that serum FGF-23 before surgery level (high vs. low) [*P *= 0.015, hazards ratio (HR) = 2.940], age (≥65 years vs. <65 years) (*P *= 0.006, HR = 3.411), diabetes (yes vs. no) (*P *= 0.011, HR = 2.703), previous stroke (yes vs. no) (*P *= 0.010, HR = 5.095), LVEF <50% (yes vs. no) (*P *= 0.023, HR = 2.733), and higher disease extent (*P *= 0.011, HR = 3.303) were all independently associated with higher cumulating MACCE rate in ULMCAD patients receiving CABG (Table 3).

**Table 3:** Multivariate Cox's proportional hazards regression analysis for cumulating MACCE rate.

Items	*P* value	HR	95% CI	
			Lower	Upper
Serum FGF-23 level before surgery (high vs. low)	0.015	2.940	1.236	6.994
Age (≥65 years vs. <65 years)	0.006	3.411	1.422	8.181
Diabetes (yes vs. no)	0.011	2.703	1.255	5.823
Previous stroke (yes vs. no)	0.010	5.095	1.486	17.472
LVEF <50% (yes vs. no)	0.023	2.733	1.147	6.512
Higher disease extent^a^	0.011	3.303	1.308	8.344

MACCE, main adverse cardiovascular and cerebrovascular events; HR, hazards ratio; CI, confidence interval; FGF-23, fibroblast growth factor-23; LVEF, left ventricular ejection fraction.

^a^ Left main only = 1, left main +1 vessel disease = 2, left main +2 vessel diseases = 3, left main +3 vessel diseases = 4.

### The ability of the combination of independent prognostic factors for estimating MACCE rate

**Figure 5:**
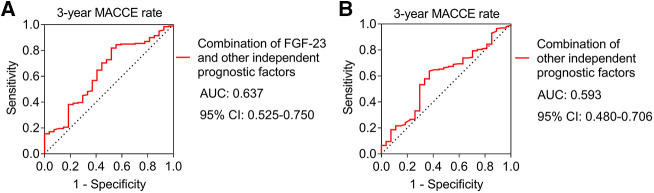
The combination of FGF-23 with other independent prognostic factors showed a certain ability to estimate the major adverse cardiac and cerebrovascular event (MACCE) rate in unprotected left main coronary artery disease patients receiving coronary artery bypass grafting. Receiver operating characteristic (ROC) curve of combination of FGF-23 and other independent factors for estimating 3-year MACCE rate (**A**); ROC curve of combination of other independent factors apart from FGF-23 for estimating 3-year MACCE rate (**B**).

The combination of FGF-23 with other independent prognostic factors had a certain ability to estimate the 3-year MACCE rate [area under the curve (AUC): 0.637, 95% confidence interval (CI): 0.525–0.750] (Figure 5A). Meanwhile, the combination of other independent prognostic factors apart from FGF-23 had no capacity to evaluate the 3-year MACCE rate in ULMCAD patients (AUC: 0.593, 95% CI: 0.480–0.706) (Figure 5B).

## Discussion

FGF-23 is a bone-derived hormone whose primary role is to regulate vitamin D metabolism and renal phosphate homeostasis; meanwhile, it has been reported that FGF-23 regulates the development of cardiovascular disease by motivating nitric oxide (NO) production, endothelial nitric oxide synthase (eNOS) expression, and cell proliferation in human aortic endothelial cells (9, 18). Moreover, a study claims that FGF-23 exhibits a higher expression in CAD patients than in non-CAD patients (19). Nevertheless, no relevant studies explore the variation of FGF-23 levels in ULMCAD patients before and after CABG. The present study observed that the FGF-23 level showed an upward trend after CABG surgery, then it decreased at discharge in ULMCAD patients. The possible reason might be that: FGF-23 was able to increase the inflammation through FGFR-mediated mechanisms, thereby reflecting the degree of inflammation to some extent (20); due to vascular injury after CABG, inflammation was higher in ULMCAD patients, which led to an increase in FGF-23 level; with the recovery from CABG surgery, the inflammation status was decreased and the abnormality of phosphate homeostasis was recovered; therefore, FGF-23 level was attenuated at discharge (21).

FGF-23 is reported to serve as a risk factor to reflect adverse cardiovascular events (10, 12, 22–24). However, no study reports the correlation between perioperative serum FGF-23 level and MACCE risk in ULMCAD patients undergoing CABG. The present study found that the preoperative FGF-23 level was independently correlated with a higher cumulating MACCE rate; meanwhile, the FGF-23 level at discharge showed a trend to be positively associated with the cumulative MACCE rate but did not reach statistical significance. The possible explanations could be that FGF-23 might facilitate the progression of atherosclerosis through several methods including regulating oxidative stress and proliferation of vascular endothelial cells, as well as promoting the infiltration of macrophages (18, 20, 25, 26). Therefore, preoperative FGF-23 could directly induce the incidence of MACCE. As a result, preoperative serum FGF-23 levels could independently predict MACCE risk in ULMCAD patients receiving CABG. Besides, the reason why the FGF-23 level at discharge was not linked to cumulative MACCE rate might be that: after CABG, FGF-23 in ULMCAD patients was disturbed by the surgery itself as well as by the recovery of trauma, therefore reducing its ability to predict MACCE risk at discharge. Moreover, this study also discovered that the 1-year, 2-year, and 3-year cumulative MACCE rates in ULMCAD patients were 5.3%, 10.1%, and 20.1%. Notably, a previous study reveals the 1-year, 2-year, and 3-year MACCE rates in ULMCAD patients are 7.1%, 11.2%, and 14.3% (27). The finding of this study was partly similar to previous data.

Heart failure is a clinical syndrome caused by abnormalities in the function or structure of the heart, clinically manifested by a lack of the heart's ability to provide adequate oxygen and blood to surrounding tissues (28). Chronic renal disease is a common disease that usually presents with symptoms of renal insufficiency at the end stage and is associated with a higher cardiovascular risk (29). The present study found that FGF-23 was positively correlated with both previous heart failure and previous chronic renal failure. The possible explanation might be that previous heart failure and chronic renal failure might have caused certain damage to the cardiovascular system; meanwhile, as mentioned above, FGF-23 was able to regulate vascular endothelial injury and inflammatory cell infiltration (18, 20, 30, 31), thereby reflecting the cardiovascular injury to some extent. Thus, FGF-23 was positively associated with previous heart failure and previous chronic renal failure.

Apart from FGF-23, some traditional prognostic factors could also predict MACCE risk in ULMCAD patients receiving CABG, including age ≥65 years, diabetes, previous stroke, LVEF <50%, and higher disease extent, which was in line with a previous study (3). The finding of this study further confirmed the prognostic value of these factors, and the ROC curve based on a combination of FGF-23 with these independent prognostic factors showed a certain ability to estimate the 3-year MACCE risk in ULMACD patients receiving CABG.

There were several limitations in this study. First, the follow-up period of this study was short; therefore, the predictive value of FGF-23 for long-term MACCE risk in ULMCAD patients receiving CABG was elusive. Second, this study only included ULMCAD patients who received CABG; whether FGF-23 also could serve as a predictive biomarker for MACCE in ULMCAD patients who received other treatments remained to be investigated. Third, since most of the ULMCAD patients receiving CABG were not local residents, it was difficult to consistently obtain their blood samples, which hampered the follow-up and the long-term recording of their serum FGF-23 levels. Fourth, the sample size of this study was relatively small; further studies could consider enrolling more ULMCAD patients receiving CABG to strengthen the findings. Fifth, to reduce the cost of the research, the ELISA detection was performed in batches; therefore, there might exist detection deviation among different batches. Sixth, although all CABGs were successful, there would be some differences in operation skills among surgeons, and these may indirectly affect the subsequent MACCE risk in ULMCAD patients receiving CABG.

In conclusion, preoperative serum FGF-23 level could independently predict MACCE risk in ULMCAD patients receiving CABG, suggesting that serum FGF-23 has potential value as a prognostic biomarker in ULMCAD management. However, further validation is needed.

## Data Availability

The original contributions presented in the study are included in the article/Supplementary Material, further inquiries can be directed to the corresponding author/s.
